# Miocardiopatias em Crianças e Adolescentes na Era da Medicina de Precisão

**DOI:** 10.36660/abc.20230154

**Published:** 2024-10-08

**Authors:** Ana Flávia Mallheiros Torbey, Raquel Germer Toja Couto, Aurea Grippa, Eduarda Corrêa Maia, Sara Aimée Miranda, Marcos Adriano Cardoso dos Santos, Elion Tavares Peres, Olimpio Patrick Silva Costa, Everton Mattos de Oliveira, Evandro Tinoco Mesquita

**Affiliations:** 1 Universidade Federal Fluminense Niterói RJ Brasil Universidade Federal Fluminense, Niterói, RJ – Brasil; 2 Programa de Pós-Graduação em Ciências Cardiovasculares Universidade Federal Fluminense Niterói RJ Brasil Programa de Pós-Graduação em Ciências Cardiovasculares da Universidade Federal Fluminense, Niterói, RJ – Brasil; 3 Universidade Federal Fluminense Hospital Universitário Antônio Pedro Niterói RJ Brasil Universidade Federal Fluminense Hospital Universitário Antônio Pedro (EBSERH), Niterói, RJ – Brasil; 4 Complexo Hospitalar de Niteroi Niterói RJ Brasil Complexo Hospitalar de Niteroi, Niterói, RJ – Brasil

**Keywords:** Cardiomiopatias, Insuficiência Cardíaca, Criança, Medicina de Precisão, Genética

## Abstract

Na infância e adolescência, as miocardiopatias apresentam características próprias e são uma importante causa de insuficiência cardíaca, arritmias, morte súbita e indicação de transplante cardíaco. O diagnóstico é um desafio na prática diária devido à sua apresentação clínica variada, etiologias heterogêneas e conhecimento limitado das ferramentas de genética clínica e molecular. Entretanto, é fundamental reconhecer os diferentes fenótipos e priorizar a busca pela etiologia. Os avanços recentes na medicina de precisão tornaram o diagnóstico molecular mais acessível, permitindo individualizar condutas terapêuticas, estratificar o prognóstico e identificar indivíduos da família que estejam em risco de desenvolver doença. O objetivo desta revisão é enfatizar as particularidades das miocardiopatias na pediatria e como o enfoque individualizado influencia a terapêutica e o prognóstico do paciente. Através de uma abordagem sistematizada, o protocolo é apresentado em cinco etapas em nosso serviço. Estas etapas incluem a avaliação clínica para determinação do fenótipo morfofuncional, identificação da etiologia, classificação, estabelecimento do prognóstico e busca por terapias personalizadas.

## Introdução

As miocardiopatias são um grupo heterogêneo de alterações estruturais, mecânicas e elétricas do miocárdio, sendo a principal causa de transplante cardíaco no primeiro ano de vida.^1,2^Embora sejam raras na pediatria, podem estar subdiagnosticadas, considerando o aumento do número de casos de insuficiência cardíaca (IC) na população pediátrica.^1,3-7^

Os avanços nas técnicas de genética molecular e cardioimagem promoveram uma mudança no conhecimento e na classificação das miocardiopatias nas últimas décadas.^8-13^ Entretanto, ainda existe uma lacuna significativa no entendimento das principais etiologias, na apresentação clínica e na abordagem terapêutica em crianças e adolescentes.^14-17^

Atualmente, no Brasil, iniciativas na abordagem das miocardiopatias estão em desenvolvimento, como a Rede Nacional de Genômica Cardiovascular - RENOMICA, um programa de pesquisa que estuda as doenças cardiovasculares hereditárias e a análise de custo-efetividade do diagnóstico genético no SUS.^18^Nosso grupo está desenvolvendo o Registro de Miocardiopatias e Miocardites em Crianças e Adolescentes na região metropolitana II do Estado do Rio de Janeiro (Registro CHARISMA), estudando a relação genótipo/fenótipo.

Este trabalho tem como objetivos descrever, através de revisão da literatura, a abordagem atualizada das miocardiopatias pediátricas diante dos principais avanços da medicina de precisão. Além disso, visa apresentar o modelo de raciocínio clínico em 5 etapas utilizado em nosso serviço, que incorpora uma abordagem personalizada nas miocardiopatias pediátricas.

## Métodos

Realizou-se uma busca nas bases de dados indexadas PUBMED, LILACS e SCIELO utilizando os termos: Cardiomiopatias/Cardiomyopathy; Criança/Children; Insuficiência cardíaca/Heart Failure; Pediatria/Pediatrics; Genética/Genetics; e Medicina de Precisão/Precision Medicine. Foram selecionados artigos publicados em inglês e português, entre os anos de 1997 e 2022.

### Epidemiologia

O impacto da IC na infância vem aumentando. As principais causas de IC pediátricas são cardiopatias congênitas, miocardiopatias e arritmias.^3,4,19,20^Em estudo publicado por Salim et al.,^21^ a miocardiopatia representou 32% dos óbitos por causas cardiovasculares em crianças com menos de um ano de idade no Brasil, destacando-se como a principal causa de morte neste subgrupo entre 2000 e 2015. No estado do Rio de Janeiro, as miocardiopatias apresentaram as maiores taxas de mortalidade proporcional anual.^22^

Estudos realizados na América do Norte, Europa (Finlândia) e Austrália apontam para uma incidência estimada de 1:100.000 pessoas por ano, com idade inferior a 20 anos, diagnosticadas com miocardiopatia.^1,14,15,23^ Esses estudos mostraram um predomínio no sexo masculino e em crianças de origem negra e aborígene.^5,14,23^ A miocardiopatia dilatada (MCD) e a miocardiopatia hipertrófica (MCH) representam a maioria dos fenótipos (cerca de 50% cada); as miocardiopatias restritiva (MCR) e não compactada (MCNC) correspondem a 5%. Entretanto, essa incidência pode variar de acordo com a faixa etária estudada. A incidência da MCH é três vezes maior entre lactentes com menos de um ano de idade. O Registro Australiano mostrou uma incidência mais elevada, de aproximadamente 10%, de MCNC quando comparada aos outros registros.^1,24^

Apesar dos estudos descritos, a real incidência das miocardiopatias na população pediátrica ainda não é conhecida. Além disso, há uma lacuna significativa no conhecimento das características epidemiológicas no Brasil e na América Latina, onde faltam estudos sobre o tema.^17,25^ Recentemente, Huertas-Quiñones et al. demonstraram que as características clínicas das miocardiopatias pediátricas em um centro de referência na Colômbia estão alinhadas com as tendências globais, sendo a MCD a mais frequente, seguida pela MCH.^17^

### Classificação

As classificações atuais baseiam-se principalmente no fenótipo morfofuncional e ressaltam a importância das bases genéticas das miocardiopatias. Atualmente, estão disponíveis as classificações da *American Heart Association* (AHA) de 2006, da *European Society of Cardiology* (ESC) de 2008 e da *World Heart Federation* (WHF) de 2013 (MOGE(S)).^1,9,12,26^

A Classificação de MOGE(S), certificada pela WHF em 2013, foi desenvolvida com o objetivo de integrar as diferentes características dos pacientes com miocardiopatias e baseia-se em fenótipo morfofuncional, órgão envolvido, origem genética ou familiar, etiologia, estágio da IC (A-D da AHA) e classe funcional (I-IV da New York Heart Association).^1,9,13^ A utilização das classes funcionais tem valor limitado nas crianças e geralmente não são utilizadas. Entretanto, para crianças com idade inferior a seis anos e quadro clínico de IC, recomenda-se utilizar a classificação modificada de Ross,^1,27,28^ conforme Tabela 1.

**Tabela 1:** – Classificação funcional para crianças com insuficiência cardíaca

Capacidade funcional (NYHA)*		Classificação de Ross para crianças e lactentes	
Classe Funcional	Descrição	Classe Funcional	Descrição
I	Sem limites à atividade física; ausência de sintomas nas atividades físicas comuns.	I	Sem limitação ou sintomas.
II	Atividades físicas de rotina causam fadiga, palpitação ou dispneia. Confortável em repouso.	II	Taquipneia leve ou sudorese durante as mamadas. Dispneia aos esforços nas crianças mais velhas, sem prejuízo ao ganho ponderal.
III	Atividades físicas menores que a de rotina causam fadiga, palpitações ou dispneia. Confortável em repouso.	III	Importante taquipneia ou sudorese durante as mamadas. Prolongamento do período de amamentação. Atraso do crescimento devido a IC.
IV	Incapacidade de realizar qualquer atividade física sem desconforto, sintomas de IC em repouso. Piora do desconforto se qualquer atividade física for realizada.	IV	Sintomas em repouso com taquipneia, retrações, gemidos e sudorese.

*New York Heart Association. Fonte: Azeka et al., 2014^27^ e Monda et al., 2021.^28^

### Abordagem dos pacientes pediátricos com miocardiopatia em cinco etapas

A abordagem das miocardiopatias pediátricas consiste em uma medicina personalizada, com protocolos padronizados que auxiliam os médicos na investigação etiológica dessas condições.^29^ Como o quadro clínico é heterogêneo, os pacientes podem ser assintomáticos ou apresentar sinais e sintomas de IC, arritmias, dor no peito, síncope ou morte súbita (MS). Portanto, para que o diagnóstico seja feito, é necessário nível de suspeição elevado.^1,2,7,30^

Inicialmente, realiza-se a avaliação clínica para confirmar o fenótipo e determinar se a miocardiopatia é uma condição isolada ou sindrômica. Geralmente, é possível observar “*red flags*” específicas que podem orientar o diagnóstico adequado (Tabela 2).^29,31^ Em seguida, busca-se a etiologia para definir um plano terapêutico e prognóstico. É importante utilizar a classificação de MOGE(S) para um diagnóstico cardiológico completo das miocardiopatias, considerando as particularidades da pediatria, especialmente em relação à classe funcional.^1,7,27,28^ Por fim, avalia-se o prognóstico e aplica-se uma terapia personalizada, individualizando condutas medicamentosas e não medicamentosas de acordo com a etiologia.^6^

**Tabela 2:** – Red Flags no exame físico e miocardiopatias

Características clínicas/laboratoriais (*red flags*)	Fenótipo da miocardiopatia	Etiologia possível
Características faciais grosseiras, opacificação das córneas, atraso do crescimento e desenvolvimento, déficit intelectual, hepatoesplenomegalia.	MCD MCH	Mucopolissacaridoses
Hipotonia, fraqueza muscular, atraso do desenvolvimento.	MCH	Doença de Pompe
Baixa estatura, atraso do desenvolvimento, hipertelorismo ocular, pescoço alado, ptose palpebral.	MCH	Síndrome de Noonan e outras RASopatias
Ataxia progressiva, ausência de reflexos.	MCH, mais raramente MCD	Ataxia Friedreich
Elevação de creatinofosfoquinase e transaminases, atraso do desenvolvimento e retinopatia.	MCH	Doença de Danon
Dor neuropática em extremidades, angioqueratomas, microalbuminúria, sintomas gastrointestinais, córnea verticilata, hipoidrose.	MCH	Doença de Fabry

MCD: miocardiopatia dilatada; MCH: miocardiopatia hipertrófica.

Apresentamos a seguir as etapas de avaliação do paciente pediátrico com suspeita de miocardiopatia que utilizamos em nosso serviço (Figura Central).

**Figura Central:**
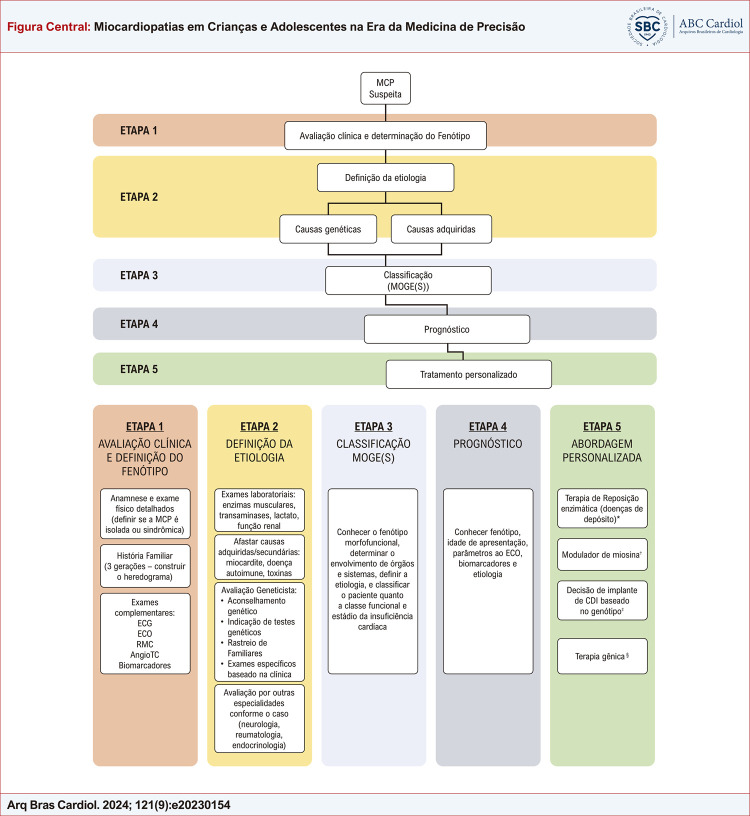
: Miocardiopatias em Crianças e Adolescentes na Era da Medicina de Precisão

Abordagem sistematizada em cinco etapas das miocardiopatias de início na infância e adolescência. A porção superior da figura mostra o fluxograma do raciocínio clínico, a porção inferior da figura traz o detalhamento de cada etapa.MCP: miocardiopatia, ECG: eletrocardiograma, ECO: ecocardiograma, RMC: ressonância magnética cardíaca, Angio TC: angiotomografia. *os principais exemplos são as doenças de Pompe, Fabry e Mucopolissacaridoses ^†^ disponível para adultos com miocardiopatia obstrutiva hipertrófica, ^‡^ algumas variantes gênicas estão mais associadas ao risco de arritmias e morte súbita, ^§^ existem em andamento estudos promissores para a distrofia muscular de Duchenne e para a doença de Danon.

#### Etapa 1

A história pessoal é de fundamental importância, destacando-se a idade do diagnóstico ou de início dos sintomas. Neonatos e lactentes apresentam, com maior frequência, erros inatos do metabolismo (EIM) e síndromes genéticas como etiologia, em comparação com escolares e adolescentes.^1,2,7,31,32^

No exame físico de lactentes e pré-escolares com MCD, é comum observar os sinais clássicos de IC.^7,33^ História de síncope, sopro cardíaco e dor torácica também podem estar presentes em todos os fenótipos.^7,8,34^ Estima-se que 40% das crianças sintomáticas desenvolvem IC tão grave que necessitam de transplante cardíaco ou evoluem para o óbito em cinco anos.^19,20,35^Nas crianças com MCH, a MS é a principal causa de óbito.^36^

É essencial realizar uma avaliação global da criança, com foco nos marcos do desenvolvimento motor e cognitivo, na presença de dismorfismos, nas alterações antropométricas e em sinais de fraqueza muscular ou envolvimento de outros órgãos e sistemas.^2,7,29^ Estima-se que cerca 10% das crianças com miocardiopatia tenham um diagnóstico de síndrome genética, e 15% das causas conhecidas são atribuídas aos EIM, o que os diferencia da população adulta.^32,37^

Na história patológica pregressa, deve-se investigar a presença de infecções recentes (respiratórias e gastrointestinais), história de tratamento oncológico, condições inflamatórias ou doença autoimune, e doenças endócrinas, descartando causas não genéticas para a miocardiopatia.^1,2,7,31^

Recomenda-se a elaboração de uma história familiar detalhada, incluindo no mínimo três gerações em formato de heredograma. Assim, é possível reconhecer o padrão de herança, realizar o aconselhamento genético e identificar indivíduos em risco de desenvolver a doença, além de fornecer informações como idade de apresentação, possíveis desfechos e variação de fenótipos em uma mesma família.^1,7,29,31,37^ É importante ressaltar que uma criança com diagnóstico de miocardiopatia genética pode ter história familiar negativa, com a presença de uma mutação *de novo*, ou ter herdado a alteração genética de um progenitor assintomático.^31,37^

O padrão de herança geralmente é autossômico dominante, mas também podem ocorrer padrões autossômicos recessivos, ligado ao X ou doença mitocondrial (padrão matrilinear), sendo mais frequentes em crianças do que em adultos.^1,2^ Alterações em genes do sarcômero são as mais comuns em crianças com MCH isolada, mas também podem ser encontradas em crianças com MCD, MCNC e MCR. Mutações também podem ocorrer em genes do citoesqueleto, membrana nuclear e desmossomos. Além disso, uma mesma variante patogênica pode causar diferentes fenótipos, e diferentes fenótipos das miocardiopatias podem ocorrer dentro da mesma família.^1,2,37,38^

**Figura 1:**
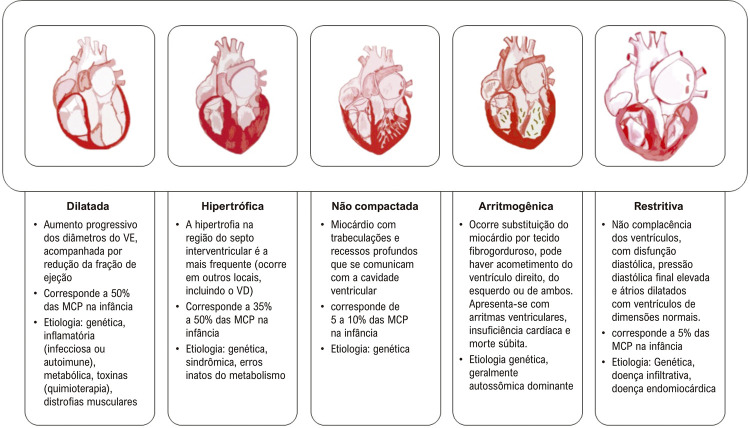
– Principais fenótipos e características das miocardiopatias pediátricas. VE: ventrículo esquerdo; MCP: miocardiopatia; VD: ventrículo direito.

Após realização de anamnese e exame físico, o fenótipo morfofuncional é determinado por meio de exames complementares de cardioimagem e testes laboratoriais.^1,2,7^A figura 1 mostra os principais fenótipos na pediatria e suas características.

Muitas vezes, devido à complexidade da miocardiopatia, é necessária uma avaliação multidisciplinar envolvendo médicos que possuam conhecimentos em cardiologia, pediatria, metabolismo, radiologia, neurologia e genética.^29,31^

1. Eletrocardiograma (ECG): de fácil acesso e baixo custo, auxilia no diagnóstico das miocardiopatias, identificando alterações como baixa voltagem do QRS, aumento de cavidades cardíacas, alterações na condução atrioventricular, repolarização ventricular, e a presença de arritmias atriais e ventriculares. Em alguns pacientes, a alteração no ECG pode ser a única manifestação fenotípica de doença miocárdica. A presença de Wolff-Parkinson-White e pré-excitação ventricular está associada a doenças de depósito, como nas mutações na PRKAG2 e na LAMP2 (doença de Danon) e doença de Pompe. Alterações progressivas na condução atrioventricular e bloqueio atrioventricular são comuns nas laminopatias, doenças mitocondriais e doenças de depósito ou infiltrativas, além de estarem presentes em doenças inflamatórias, como miocardite de células gigantes e sarcoidose.^1,29,31,37,39^
2. Ecocardiograma: fornece informações sobre a anatomia, ajudando a confirmar ou excluir a presença de doença congênita e determinando as dimensões das cavidades e paredes cardíacas, que devem ser expressas em z-escores. Além disso, avalia as funções sistólica e diastólica. O uso do *Strain* (longitudinal, circunferencial e radial) é sensível para disfunção sistólica, e permite o diagnóstico precoce de miocardiopatia induzida por quimioterapia e de miocardiopatias hereditárias, principalmente nos fenótipos de MCH e MCNC como demonstrado em revisão sistemática realizada por Dorobantu et al.^1,7,8,27,39,40^ Ainda, a observação de características do miocárdio auxilia no diagnóstico de MCNC.^41^ A presença de hipertrofia concêntrica do VE maior que 3 cm geralmente está associada a doenças de depósito, como a doença de Pompe nos lactentes e a doença de Danon em adolescentes.^1,31^
3. Holter de 24 horas e Teste Ergométrico: detectam a presença de arritmias atriais e ventriculares, auxiliam na estratificação de risco para MS, e são fundamentais neste grupo de pacientes.^1,20^
4. Ressonância magnética cardíaca (RMC): auxilia na determinação do fenótipo morfológico e estuda as funções sistólica e diastólica. A presença de edema miocárdio, hiperemia e fibrose de padrão não isquêmico está associada à miocardite, enquanto a presença de substituição de tecido fibrogorduroso pode indicar miocardiopatia arritmogênica. A fibrose (realce tardio) também auxilia no diagnóstico das doenças neuromusculares e em pacientes submetidos a terapia com antracíclicos.^1,7,8,27,31^
5. Tomografia cardíaca: indicada para pacientes com janela ecocardiográfica difícil e com contraindicações para a RMC. É indispensável no estudo das coronárias e da anatomia cardíaca. Pode revelar a presença de tecido gorduroso, característico da miocardiopatia arritmogênica.^1,8^
6. Biomarcadores: O peptídeo natriurético cerebral (BNP) e o fragmento aminoterminal de seu precursor (NT-ProBNP) são produzidos em resposta à sobrecarga de volume, pressão e tensão na parede ventricular. Sua dosagem auxilia no prognóstico de crianças com miocardiopatias e IC; com níveis mais elevados, indicam maior risco de piores desfechos em crianças com MCD. Também auxiliam no diagnóstico diferencial entre cardiomiopatia restritiva (níveis mais elevados) e pericardite constritiva. Em pacientes submetidos a tratamento com quimioterápicos cardiotóxicos, sua elevação indica precocemente a lesão e disfunção miocárdica.^1,7,27,39,42^A troponina eleva-se em crianças com miocardiopatias, embora não haja associação com prognóstico. A elevação geralmente ocorre na MCD, decorrente de miocardiopatia inflamatória como as miocardites.^1,27,39^ Em pacientes com miocardiopatia arritmogênica, a troponina pode aumentar na “fase quente”, conforme descrito por Bariani et al., onde há apresentação clínica com dor torácica e elevação da troponina na ausência de alterações coronarianas.^43^ A dosagem das enzimas musculares, bem como a avaliação da função hepática e renal, é fundamental para a análise de comprometimento multi-orgânico.^29^
7. Cateterismo cardíaco: indicado para condições específicas. Determina a resistência vascular pulmonar, avalia a circulação coronária, afasta anomalias coronárias, confirma a fisiologia da miocardiopatia restritiva (MR) e realiza biópsia endomiocárdica.^1,3,27,33^

#### Etapa 2

A Etapa 2 consiste na identificação da etiologia. Embora o fenótipo morfofuncional seja semelhante aos observados na população adulta, a prevalência das etiologias difere entre crianças e adolescentes. Doenças neuromusculares, metabólicas, mitocondriais e outras síndromes genéticas são causas importantes, principalmente em lactentes e pré-escolares.^1,2,7,25,29,42^

Na infância, a investigação metabólica e a dosagem de enzimas musculares ajudam a esclarecer algumas condições. A elevação de creatinofosfoquinase pode indicar doença mitocondrial e doença de Danon em pacientes com MCH, enquanto nos pacientes com MCD, a elevação das enzimas musculares está associada a distrofinopatias, sarcoglicanopatias, laminopatias, distrofia miotônica e desminopatias. Já a presença de acidose lática e a elevação de transaminases apontam para doença mitocondrial, enquanto os pacientes com doença de Fabry podem apresentar proteinúria.^31,37^

Os EIM correspondem a cerca de 15% das causas conhecidas de miocardiopatias, sendo etiologias frequentes das MCH e MCD.^2,7,25,32^ Exemplos incluem as doenças de depósito do glicogênio, como a doença de Pompe e a doença de Danon, as doenças de depósito lisossomal, como as mucopolissacaridoses (MPS), e a doença de Fabry, que representa os distúrbios do metabolismo dos glicoesfingolipídeos.^32^

A triagem neonatal para EIM realizada rotineiramente não cobre todos os testes diagnósticos necessário, o que pode levar ao subdiagnóstico desses distúrbios associados à miocardiopatia devido à falta de conhecimento.^32^A doença de Pompe deve ser afastada em lactentes com MCH acompanhada de hipotonia, através da dosagem da atividade da enzima alfa glicosidase ácida. A suspeita de doença de Fabry exige a dosagem da enzima alfa galactosidase A. Para as MPS, é importante dosar os glicosaminoglicanos urinários, enquanto a doença de Danon é causada pela deficiência de proteína de membrana 2 associada ao lisossomo (LAMP2).^1,2,7,32^ O diagnóstico etiológico deste grupo de doenças é fundamental, pois pode permitir tratamento específico e requer abordagem multidisciplinar.^25,38,44^

A síndrome de Noonan é a principal causa genética de MCH em crianças com menos de um ano de idade, sendo acompanhada de elevado risco de mortalidade precoce.^1,32^

A avaliação genética consiste em uma abordagem sistemática que inclui uma história familiar detalhada (ETAPA 1), aconselhamento genético e a realização de testes genéticos específicos, quando indicados.^1,42^

O geneticista clínico afasta condições sindrômicas e indica quais exames genéticos devem ser solicitados. É fundamental compreender os aspectos ético-legais que variam em cada país e os impactos psicossociais que podem ser gerados. Assim, essas questões devem ser discutidas durante o aconselhamento. Nesse momento, o médico geneticista também aponta quais familiares estariam em risco de desenvolver a doença, orientando o rastreio clínico e genético em cascata destes parentes. Os consensos atuais recomendam que a investigação genética seja realizada em crianças e adultos com diagnóstico de miocardiopatia.^37,45-49^

#### Exames genéticos

Durante o aconselhamento genético, a família é informada sobre os possíveis resultados da investigação: (1) resultado conclusivo, onde é identificada uma variante patogênica ou provavelmente patogênica que justifique o fenótipo em questão, (2) resultado negativo, onde nenhuma variante que justifique a miocardiopatia é detectada, ou (3) resultado inconclusivo, onde uma variante identificada é classificada como de significado incerto (VUS). Nesse caso, é importante manter o acompanhamento e reavaliar o potencial patogênico da variante futuramente.^1,42,50,51^É essencial que a interpretação desses achados seja realizada por profissional com treinamento em cardiogenética.

A probabilidade de um exame genético ser positivo depende não só do tipo de miocardiopatia em questão, mas também das características clínicas que podem indicar doenças associadas específicas, como no caso das distrofias musculares. Para pacientes adultos, a taxa de positividade do exame genético pode variar de 60% a 70% para a MCH, de 30% a 40% para a MCD e de 50% a 60% para a miocardiopatia arritmogênica do ventrículo direito.^52^ A determinação da etiologia genética das miocardiopatias pediátricas, por outro lado, ocorre em torno de 32 a 39%.^6,53^

A maioria das miocardiopatias apresenta alterações monogênicas, sendo indicada a realização da técnica de sequenciamento genético de nova geração (NGS). Existem painéis específicos para determinados fenótipos, sendo estes exames indicados para indivíduos com um fenótipo bem definido e isolado de miocardiopatia. Entretanto, quando houver comprometimento multissistêmico, presença de dismorfias ou suspeita de miocardiopatia sindrômica, a análise completa do exoma é útil. Para estudar a presença de uma variante já identificada em casos de rastreio genético familiar, utiliza-se a técnica de Sanger.^49,50,54^

Embora raras, alterações cromossômicas podem estar associadas às miocardiopatias, como na síndrome de Pallister-Killian, uma tetrassomia de 12p que pode cursar com MCH, e na MCR com anomalia do cromossomo 6. Para o diagnóstico desses casos, o cariótipo de sangue periférico é o exame preferencial.^55,56^

O alto custo dos exames genéticos e a necessidade de profissionais treinados em cardiogenética para a correta indicação e interpretação dos resultados limitam a sua utilização. Além disso, com a disseminação do uso do exoma, é possível a detecção de achados secundários ou incidentais que não são relacionados com a doença cardiovascular a ser investigada, como variantes patogênicas em genes relacionados ao desenvolvimento de câncer. É fundamental que a possibilidade de tais achados seja discutida com o paciente antes da coleta do exame, e que o paciente dê consentimento para receber ou não tais resultados.^51^

#### Rastreio familiar

Ocorre quando um indivíduo da família do probando é avaliado devido à história familiar de uma miocardiopatia previamente definida, independentemente de apresentar ou não fenótipo/sintomas clínicos. Aqueles com padrão de herança autossômica dominante têm 50% de probabilidade de transmitir a variante para seus filhos.^37,50,57^

O rastreio clínico é recomendado para todo parente de primeiro grau que esteja sob risco de desenvolver a miocardiopatia, mesmo que assintomático. Portanto, a avaliação com anamnese, exame físico, ECG e ecodoppler colorido deve ser realizada para identificar o fenótipo. A Sociedade Americana de Insuficiência Cardíaca (*Heart Failure Society of America*) recomenda a triagem clínica anual para crianças até 5 anos de idade, a cada 1 a 2 anos para crianças entre 6 e 12 anos de idade, e a cada 1 a 3 anos para os adolescentes entre 13 e 19 anos. A triagem deve continuar na vida adulta com avaliações regulares a cada 3 a 5 anos.^50^

Rastreio genético: uma vez que uma variante patogênica seja identificada, ela poderá ser pesquisada através do *rastreio em cascata* nos parentes de primeiro grau. Caso a variante seja detectada, o indivíduo deverá manter a investigação clínica em busca do fenótipo e receber aconselhamento genético. Diante da presença de um genótipo positivo, não é possível determinar se uma miocardiopatia irá se manifestar clinicamente, caracterizando que a penetrância pode ser variável, assim como a expressividade, onde diferentes fenótipos podem ocorrer associados a uma mesma variante. Caso a variante não seja identificada nos parentes estudados, não há necessidade de manter rastreio fenotípico com realização de exames cardiovasculares rotineiros.^37,50,57^

É comum que pais e responsáveis temam que seus filhos sejam estigmatizados, enfrentem discriminação e sofram danos psicológicos caso testem positivo para uma variante genética familiar. Entretanto, os benefícios do conhecimento do genótipo são potenciais e incluem a capacidade de esclarecer quais crianças precisam cuidados cardíacos, orientação na participação de atividades esportivas e redução da preocupação quando o teste é negativo.^50,57,58^

#### Etapa 3

Nesta etapa, já temos o fenótipo morfofuncional e a etiologia definidos. Com base nessas informações, é possível utilizar a classificação de MOGE(S).^1,9^

#### Etapa 4

Em seguida, é fundamental determinar o prognóstico. Ele pode variar e relaciona-se diretamente com o fenótipo, genótipo, idade do paciente, presença de arritmias cardíacas, elevação de biomarcadores e classe funcional.^1,2,35^

A MCD apresenta prognóstico reservado, onde cerca de 30% dos pacientes evoluem para óbito ou necessitam de transplante cardíaco após três anos da doença, devido a IC avançada ou arritmias.^2,34,39^Estudos mostram que a idade ao diagnóstico (inferior a 1 e acima de 12 anos), alterações nas funções sistólica e diastólica do VE, além do diâmetro diastólico final do VE são preditores de progressão da doença, eventos adversos, transplante cardíaco e óbito. A elevação do NT-proBNP/BNP também está associada a piores desfechos.^35,39,44,59-61^A sobrevida, sem necessidade de transplante cardíaco, pode variar de 60% a 75% dentro de 5 anos após o diagnóstico.^7,33,60^ Um terço dos pacientes pode recuperar a função ventricular, ocorrendo com maior frequência nos pacientes com miocardite comprovada por biopsia, com menor dimensão diastólica final do VE e maior espessura da parede septal. A MCD é a principal indicação de transplante cardíaco em crianças, com uma boa taxa de sobrevida de 94% no primeiro ano do transplante.^1,2,7,39,62^

A MCH apresenta um prognóstico pior em crianças no primeiro ano de vida, especialmente quando os EIM e síndromes de malformação são a etiologia.^1,2,7,63^ A presença de desnutrição e IC no diagnóstico agrava o prognóstico, conforme demonstrado nos dados obtidos do Registro Pediátrico Norte Americano de Miocardiopatias.^63^

Os pacientes com MCH podem ter diferentes taxas de sobrevida de acordo com a etiologia subjacente. A pior relaciona-se aos EIM, com 42% em cinco anos, seguido das síndromes de malformação com 74%. Quando o diagnóstico é realizado após um ano de idade e a causa não é determinada, a sobrevida chega a 94% em cinco anos.^2,7,63^

A principal causa de óbito em crianças com MCH é a MS, sendo mais comum do que em adultos.^64^ Os principais fatores de risco associados à MS incluem síncope inexplicada, espessura máxima da parede do VE, diâmetro AE, gradiente na via de saída do VE e taquicardia ventricular não sustentada. A história familiar de MS parece não estar associada a risco aumentado na infância.^65,66^

Dados do estudo ShaRe (*The Sarcomeric Human Cardiomyopathy Registry)* mostraram que, quando a presença de variantes sarcoméricas são detectadas em crianças com MCH, há maior risco de desenvolver arritmias ventriculares e necessidade de tratamento avançado para a IC. Está associado a um risco elevado de 67% para a ocorrência de desfechos cardíacos, com risco duas vezes maior de desenvolver IC.^67^ Variantes nos genes MYBPC3 e MYH7 relacionam-se a arritmias malignas.^64^ As calculadoras de risco para a população pediátrica estão sendo validadas para serem utilizadas em menores de 16 anos (*HCM Risk-Kids* https://hcmriskkids.org/).^36,66^

Pacientes com o fenótipo de MR caracterizam-se por possuir um prognóstico desfavorável, sendo o pior de todas as miocardiopatias. A sobrevida em 5 anos é de 68%, e a presença de IC e redução da fração de encurtamento complica o prognóstico. Geralmente, são referidos aos programas de transplante cardíaco precocemente.^1,2,7^

Na MCNC, o prognóstico está mais relacionado à apresentação clínica do que ao fenótipo. Pacientes assintomáticos e fenótipo isolado de MCNC apresentam melhor evolução.^1,2,7,41,68^

#### Etapa 5

Nesta última etapa, deve-se buscar o que há disponível para o tratamento das miocardiopatias pediátricas, à luz da aplicação da medicina de precisão.

O tratamento personalizado do paciente e sua família torna-se possível a partir da confirmação do fenótipo. Pacientes com MCH e miocardiopatia arritmogênica são proibidos de atividade física competitiva e podem ser candidatos a cardiodesfibrilador implantável (CDI) como prevenção primária de MS. Conhecer o genótipo do paciente e correlacionar com a história natural e progressão da doença permitem a tomada de decisões terapêuticas importantes, como a priorização do transplante cardíaco ou o uso de terapia de reposição enzimática (TRE).^1,2,6,7,29,32,50^

Atualmente, muitos dos EIM são tratáveis usando abordagens que visam a fisiopatologia envolvida no desenvolvimento da doença, sendo possível, em alguns casos, a reversão da miocardiopatia.^2,7,23,32^Na deficiência sistêmica de carnitina, por exemplo, há melhora da miocardiopatia dilatada com a reposição de altas doses de carnitina.^32^

O desenvolvimento de terapias específicas tem progredido devido ao avanço nas pesquisas clínicas. A TRE realizada na doença de Pompe melhora a hipertrofia miocárdica, com melhores resultados quando o tratamento é iniciado precocemente.^2,7,23,32^ Outras doenças do depósito lisossomal, que são associadas à miocardiopatia, incluindo doença de Gaucher, Doença de Fabry, mucopolissacaridoses I, II, IV e VI, são tratadas com TRE ou transplante de medula óssea, obtendo-se bons resultados. A TRE também tem sido bem-sucedida na mutação PRKAG2.^7,32,38,69^

Ao se identificar mutações em genes como DES, SCN5A, FLNC e LMNA, os quais apresentam risco proeminente de arritmias fatais, o uso precoce do CDI pode ser benéfico, já que a MS pode ser o primeiro sintoma e preceder o desenvolvimento da miocardiopatia.^35-37^

A decisão do implante do CDI ainda é um desafio entre pacientes pediátricos. Há uma calculadora de risco (*HCM Risk-Kids* https://hcmriskkids.org/) disponível que pode auxiliar na avaliação individualizada destes pacientes.^35,66^

Os moduladores de miosina são uma nova classe de agentes farmacêuticos que estão sendo desenvolvidos para tratar pacientes com uma variedade de miocardiopatias. Uma estratégia para diminuir a taxa de ATPase da miosina provou, recentemente, ser eficaz para o tratamento da miocardiopatia hipertrófica obstrutiva em pacientes adultos, o fármaco mavacamten.^70,71^

A terapia gênica tem como objetivo o tratamento da etiologia a nível molecular. Recentemente, Bains et al.^72^ publicaram uma revisão acerca de sua aplicação nas doenças cardiovasculares monogênicas, dentre elas as miocardiopatias. É possível realizar edição, silenciamento e substituição gênica.

A terapia de substituição genética tem aplicação terapêutica quando há uma variante com perda de função. Assim, objetiva-se introduzir o gene funcionante na célula através de um vetor, geralmente o adenovírus. Atualmente, há um ensaio clínico deste tipo de tratamento para a miocardiopatia por variante patogênica em LAMP2, que caracteriza a doença de Danon. Mutações em FLNC, MYBPC3, TTN, DSP, PKPs, BAG3 e LMNA são exemplos potenciais onde pode haver aplicação desta terapia.^71,72^

A terapia de silenciamento genético tem por objetivo reduzir a expressão de um gene com variante patogênica onde haja produção de uma proteína alterada. Tratamentos específicos para a distrofia muscular de Duchenne e amiloidose por transtirretina (ATTR) têm apresentado resultados promissores.^71,72^

A edição genética ocorre com o uso da tecnologia da CRISPR-Cas9, sendo possível uma clivagem de DNA direcionada num local preciso no genoma, determinado pela sequência única de um RNA guia, corrigindo a mutação presente no gene.^72^

Os avanços da medicina translacional e de precisão tornam possível que o tratamento das miocardiopatias esteja muito próximo à etiologia genética, corrigindo a causa ou as alterações funcionais celulares. Existem aplicações promissoras destas terapias; entretanto, ainda possuem custo elevado, o que limita o seu uso.^73-76^

## Conclusões

As miocardiopatias diagnosticadas na infância e adolescência constituem um grupo muito complexo de etiologias, fenótipos, apresentações clínicas e prognóstico diversos. A abordagem em etapas, de modo a padronizar o raciocínio clínico, assegura a determinação do fenótipo morfofuncional, a investigação da etiologia pelos métodos disponíveis, a classificação adequada do paciente para a definição do prognóstico e aplicação individualizada das terapias vigentes, específicas para cada caso.

Esta abordagem sistematizada confere a importância do conhecimento da etiologia na definição de planos terapêuticos individualizados, levando a um melhor prognóstico. Portanto, construímos uma abordagem sistematizada com cinco etapas, conectando a classificação de MOGE(S) e reforçando o papel do geneticista clínico.

O estudo do impacto da incorporação dos testes genéticos nas miocardiopatias no SUS poderá promover importantes avanços no cuidado destes pacientes, sendo fundamental a pesquisa clínica nesta área.
