# Supplementary material for: Miocardiopatias em Crianças e Adolescentes na Era da Medicina de Precisão
Source: Arq Bras Cardiol. 2024 Oct 8;121(9):e20230154. [Article in Portuguese] doi: 10.36660/abc.20230154 (PMC11634207; doi:10.36660/abc.20230154)
Supplement: Translation

# Translation: Cardiomyopathy in Children and Adolescents in the Era of Precision Medicine

**DOI:** 10.36660/abc.20230154i

**Authors:** Ana Flávia Mallheiros Torbey, Raquel Germer Toja Couto, Aurea Grippa, Eduarda Corrêa Maia, Sara Aimée Miranda, Marcos Adriano Cardoso dos Santos, Elion Tavares Peres, Olimpio Patrick Silva Costa, Everton Mattos de Oliveira, Evandro Tinoco Mesquita

**Affiliations:** 1 Brazil Universidade Federal Fluminense, Niterói, RJ – Brazil; 2 Brazil Programa de Pós-Graduação em Ciências Cardiovasculares da Universidade Federal Fluminense, Niterói, RJ – Brazil; 3 Brazil Universidade Federal Fluminense Hospital Universitário Antônio Pedro (EBSERH), Niterói, RJ – Brazil; 4 Brazil Complexo Hospitalar de Niteroi, Niterói, RJ – Brazil

**Keywords:** Cardiomyopathies, Heart Failure, Child, Precision Medicine, Genetics

## Abstract

In childhood and adolescence, cardiomyopathies have their own characteristics and are an important cause of heart failure, arrhythmias, sudden death, and indication for heart transplantation. Diagnosis is a challenge in daily practice due to its varied clinical presentation, heterogeneous etiologies, and limited knowledge of tools related to clinical and molecular genetics. However, it is essential to recognize the different phenotypes and prioritize the search for the etiology. Recent advances in precision medicine have made molecular diagnosis accessible, which makes it possible to individualize therapeutic approaches, stratify the prognosis, and identify individuals in the family who are at risk of developing the disease. The objective of this review is to emphasize the particularities of cardiomyopathies in pediatrics and how the individualized approach impacts the therapy and prognosis of the patient. Through a systematized approach, the five-stage protocol used in our service is presented. These stages bring together clinical evaluation for determining the morphofunctional phenotype, identification of etiology, classification, establishment of prognosis, and the search for personalized therapies.

## Introduction

Cardiomyopathies are a heterogeneous group of structural, mechanical, and electrical alterations of the myocardium and the main cause of heart transplantation in the first year of life.^1,2^ While uncommon in pediatrics, they may be overlooked, considering the rising number of cases of heart failure (HF) in the pediatric population.^1,3-7^

Advances in molecular genetics and cardiac imaging techniques have promoted changes in the knowledge and classification of cardiomyopathies in recent decades.^8-13^ However, there is still a significant gap in knowledge of the main etiologies, clinical presentation, and therapeutic approach in children and adolescents.^14-17^

Brazil is currently advancing initiatives to address cardiomyopathies, including the Brazilian National Cardiovascular Genomics Network - RENOMICA, which investigates hereditary cardiovascular diseases and assesses the cost-effectiveness of genetic diagnosis within the Brazilian Unified Health System (SUS).18 Our team is establishing the Registry of Cardiomyopathies and Myocarditis in Children and Adolescents in Metropolitan Region II of the State of Rio de Janeiro (CHARISMA Registry), which will focus on the genotype/phenotype relationship.

This work aims to describe, through a literature review, the updated approach to pediatric cardiomyopathies in light of the major advances in precision medicine. It also presents the 5-step clinical reasoning model employed in our service, which incorporates a personalized approach to pediatric cardiomyopathies.

## Methods

The research was conducted in the indexed databases PUBMED, LILACS and SCIELO using the terms: Cardiomiopatias/Cardiomyopathy; Criança/Children; Insuficiência cardíaca/Heart Failure; Pediatria/Pediatrics; Genética/Genetics; e Medicina de Precisão/Precision Medicine. Articles published in English and Portuguese between 1997 and 2022 were selected.

### Epidemiology

The impact of HF in childhood has increased. The main causes of pediatric HF are congenital heart disease, cardiomyopathies and arrhythmias.3,4,19,20 In a study by Salim et al.,^21^ cardiomyopathy accounted for 32% of deaths from cardiovascular origin in children under one year of age in Brazil, standing out as the main cause of death in this subgroup between 2000 and 2015. In the state of Rio de Janeiro, cardiomyopathies had the highest annual proportional mortality rates.^22^

Studies conducted in North America, Europe (Finland), and Australia show an estimated incidence of 1:100,000 people per year under the age of 20 years diagnosed with cardiomyopathy.^1,14,15,23^ These studies revealed a predominance in males and children of black and Aboriginal origin.^5,14,23^ Dilated cardiomyopathy (DCM) and hypertrophic cardiomyopathy (HCM) represent the majority of phenotypes (approximately 50% each), with restrictive cardiomyopathy (RCM) and non-compaction cardiomyopathy (NCCM) accounting for 5%. However, this incidence may vary according to the age group investigated. The incidence of HCM is three times higher among infants under one year of age. The Australian Registry showed a higher incidence of NCCM of approximately 10% when compared to other registries.^1,24^

Despite the results of the studies described, the real incidence of cardiomyopathies in the pediatric population is yet unknown. Moreover, there is a large knowledge gap regarding the epidemiological characteristics in Brazil and Latin America, where there is a lack of studies on the subject.^17,25^Huertas-Quiñones et al. have recently demonstrated that the clinical characteristics of pediatric cardiomyopathies in a reference center in Colombia agree with global trends, with DCM being the most frequent, followed by HCM.^17^

### Classification

Current classifications are based mainly on the morphofunctional phenotype and emphasize the importance of the genetic basis of cardiomyopathies. The 2006 American Heart Association (AHA) classification, the 2008 European Society of Cardiology (ESC) classification, and the 2013 World Heart Federation (WHF) classification (MOGE(S)) are currently available.^1,9,12,26^

The MOGE(S) classification, as endorsed by the WHF in 2013, was designed to integrate diverse aspects of cardiomyopathy patients, as it considers the morphofunctional phenotype, affected organ, genetic or familial origin, etiology, HF stage (AHA A-D), and functional class (New York Heart Association I-IV).^1,9,13^ The utility of functional classes is limited in children and is typically not applied. However, for children under six years of age with clinical signs of HF, the modified Ross classification^1,27,28^ is recommended, as shown in Table 1.

**Table 1:** – Functional classification for children with heart failure

Functional capacity (NYHA)*		Ross’ classification for children and infants	
Functional Class	Description	Functional Class	Description
I	No limitation to physical activity; absence of symptoms during common physical activities.	I	No limitations or symptoms.
II	Routine physical activities cause fatigue, palpitations or dyspnea. Comfortable at rest.	II	Slight tachypnea or sweating during breastfeeding. Dyspnea on exertion in older children, without compromising weight gain.
III	Minor routine physical activities cause fatigue, palpitations or dyspnea. Comfortable at rest.	III	Significant tachypnea or sweating during breastfeeding. Prolongation of the breastfeeding time. Growth delay due to HF.
IV	Inability to perform any physical activity without discomfort, HF symptoms at rest. Worsening of discomfort signs when any physical activity is performed.	IV	Symptoms at rest include tachypnea, retractions, groaning, and sweating.

*New York Heart Association. Source: Azeka et al., 2014^27^ Monda et al., 2021.^28^

### A five-step approach to pediatric patients with cardiomyopathy

The approach to pediatric cardiomyopathies consists of personalized medicine, with standardized protocols that assist physicians in the etiological investigation of these conditions.^29^ As the clinical picture is heterogeneous, patients may be asymptomatic or present with signs and symptoms of HF, arrhythmias, chest pain, syncope or sudden death. Therefore, a high level of suspicion is required for a proper diagnosis to be made.^1,2,7,30^

Initially, a clinical evaluation is performed to confirm the phenotype and determine whether the cardiomyopathy is an isolated or syndromic condition. Generally, specific “red flags” can be seen, which can lead to an appropriate diagnosis (Table 2).^29,31^ Next, the etiology is sought to define a therapeutic and prognostic plan. Using the MOGE(S) classification is crucial for a comprehensive cardiological diagnosis of cardiomyopathies, considering the specificities of pediatrics, particularly regarding the functional class.^1,7,27,28^ Ultimately, prognosis is evaluated, and personalized therapy is implemented, tailoring drug and non-drug interventions based on the underlying etiology.^6^

**Table 2:** – Red Flags in the physical examination and cardiomyopathies

Clinical/laboratory characteristics (red flags)	Cardiomyopathy phenotype	Possible etiology
Coarse facial features, corneal opacification, growth and development delay, intellectual deficit, hepatosplenomegaly.	DCM HCM	Mucopolysaccharidoses
Hypotonia, muscle weakness, developmental delay.	HCM	Pompe disease
Short stature, developmental delay, ocular hypertelorism, webbed neck, eyelid ptosis.	HCM	Noonan syndrome and other RASopathies
Progressive ataxia, absence of reflexes.	HCM, more rarely DCM	Friedreich ataxia
Elevated creatine phosphokinase and transaminases, developmental delay and retinopathy.	HCM	Danon disease
Neuropathic pain in the extremities, angiokeratomas, microalbuminuria, gastrointestinal symptoms, cornea verticillata, hypohidrosis.	HCM	Fabry disease

DCM: dilated cardiomyopathy; HCM: hypertrophic cardiomyopathy.

Below are shown the stages of evaluation of pediatric patients with suspected cardiomyopathy employed in our service (Central Illustration).

**Central Illustration:** : Cardiomyopathy in Children and Adolescents in the Era of Precision Medicine

Systematic approach in five stages for cardiomyopathies starting in childhood and adolescence. The upper part of the figure shows the flowchart of clinical reasoning, while the lower part provides details of each stage. MCP: cardiomyopathy, ECG: electrocardiogram, ECHO: echocardiogram, CMR: cardiac magnetic resonance, CT Angio: computed tomography angiography. *Main examples include Pompe disease, Fabry disease, and mucopolysaccharidoses. † Available for adults with hypertrophic obstructive cardiomyopathy. ‡ Some genetic variants are more associated with the risk of arrhythmias and sudden death. § Promising studies are underway for Duchenne muscular dystrophy and Danon disease.

#### Step 1

Personal history is crucial, as it highlights the age of diagnosis or symptoms onset. Neonates and infants present more frequently inborn errors of metabolism (IEM) and genetic syndromes as etiology when compared to schoolchildren and adolescents.^1,2,7,31,32^

During physical examination of infants and preschoolers with DCM, the classic signs of HF are commonly observed.^7,33^ History of syncope, heart murmur, and chest pain may also be present in all phenotypes.^7,8,34^ It is estimated that 40% of symptomatic children develop HF of such severity that they require heart transplantation or die within five years.^19,20,35^ In children with HCM, sudden death (SD) is the main cause of death.^36^

A global assessment of the child is essential, focusing on the motor and cognitive development milestones, as well as any dysmorphisms, anthropometric changes, signs of muscle weakness or involvement of other organs and systems.^2,7,29^ It is estimated that approximately 10% of children with cardiomyopathy have a genetic syndrome diagnosis, and 15% of the known causes are attributed to IEM, which differentiates them from the adult population.^32,37^

Previous pathological history analysis must include recent infections (respiratory and gastrointestinal), history of oncological treatment, inflammatory conditions or autoimmune disease and endocrine diseases, thereby ruling out non-genetic causes for cardiomyopathy.^1,2,7,31^

A detailed family history analysis, including at least three generations to build the genogram, is recommended. Therefore, it is possible to determine the inheritance pattern and provide genetic counseling, identifying individuals at risk of developing the disease. Additionally, information such as age of onset, potential outcomes, and variation of phenotypes within the same family can be provided.^1,7,29,31,37^ It is worth noting that a child diagnosed with genetic cardiomyopathy may lack a positive family history, either due to a de novo mutation or inheritance from an asymptomatic parent carrying the genetic alteration.^31,37^

The inheritance pattern is usually autosomal dominant; however, autosomal recessive patterns, X-linked or mitochondrial disease (matrilineal pattern) also occur and are more frequent in children than adults.^1,2^ Mutations in sarcomere genes are prevalent in children diagnosed with isolated HCM, but can also be present in those with DCM, NCCM, and RCM. Mutations may also affect genes related to the cytoskeleton, nuclear membrane, and desmosomes. Furthermore, the same pathogenic variant can lead to various phenotypes, and different types of cardiomyopathies can manifest within the same family.^1,2,37,38^

After performing anamnesis and physical examination, the morphofunctional phenotype is determined using complementary cardiac imaging exams and laboratory tests.^1,2,7^Figure 1 shows the main phenotypes in pediatric populations and their characteristics.

**Figure 1:** – Main phenotypes and characteristics of pediatric cardiomyopathies. LV: left ventricle; CMP: cardiomyopathy; RV: right ventricle.

Due to the complexity of cardiomyopathy, a multidisciplinary evaluation is often required, with physicians specializing in cardiology, pediatrics, metabolism, radiology, neurology, and genetics.^29,31^

1. Electrocardiogram (ECG): a readily available and cost-effective diagnostic tool that aids in identifying cardiomyopathies by detecting changes such as low QRS voltage, cardiac chamber enlargement, alterations in atrioventricular conduction, ventricular repolarization, as well as the presence of atrial and ventricular arrhythmias. In some patients, ECG changes may represent the sole phenotypic manifestation of myocardial disease. Conditions like Wolff-Parkinson-White syndrome and ventricular pre-excitation are associated with storage diseases such as mutations in PRKAG2 and LAMP2 (Danon disease) and Pompe disease. Progressive alterations in atrioventricular conduction and atrioventricular block are frequently observed in laminopathies, mitochondrial diseases, and storage or infiltrative disorders, as well as in inflammatory conditions like giant cell myocarditis and sarcoidosis.^1,29,31,37,39^
2. Echocardiogram: provides anatomical information, ruling out or confirming congenital disease, determining the dimensions of the heart chambers and their walls, which should be expressed in z-scores. Additionally, it assesses systolic and diastolic functions. The use of Strain (longitudinal, circumferential, and radial) is sensitive to systolic dysfunction and provides early diagnosis of chemotherapy-induced cardiomyopathy, as well as hereditary cardiomyopathies, especially in the HCM and NCCM phenotypes, as demonstrated in a systematic review by Dorobantu et al.^1,7,8,27,39,40^ Furthermore, observation of myocardial characteristics helps in the diagnosis of NCCM.^41^ The presence of LV concentric hypertrophy greater than 3 cm is generally related to storage diseases such as Pompe disease in infants and Danon disease in adolescents.^1,31^
3. 24-hour Holter and Exercise Test: detect the presence of atrial and ventricular arrhythmias, aid in risk stratification for SD, and are essential for this group of patients.^1,20^
4. Cardiac magnetic resonance imaging (CMRI): aids in determining the morphological phenotype and studies systolic and diastolic function. The presence of myocardial edema, hyperemia, and non-ischemic fibrosis correlates with myocarditis, while fibrofatty tissue replacement is indicative of arrhythmogenic cardiomyopathy. Fibrosis (delayed enhancement) also helps in diagnosing neuromuscular diseases and in patients undergoing anthracycline therapy.^1,7,8,27,31^
5. Cardiac tomography: indicated for patients with difficult echocardiographic windows and contraindications for CMRI. It is essential in the study of the coronary arteries and cardiac anatomy. It can reveal the presence of fatty tissue, a hallmark of arrhythmogenic cardiomyopathy.^1,8^
6. Biomarkers: Brain natriuretic peptide (BNP) and the amino-terminal fragment of its precursor (NT-proBNP) are produced in response to volume overload, pressure and tension in the ventricular wall. Its measurement helps in the prognosis of children with cardiomyopathies and HF; higher levels indicate a greater risk of worse outcomes in children with DCM. It also helps in the differential diagnosis between restrictive cardiomyopathy (higher levels) and constrictive pericarditis. In patients undergoing treatment with cardiotoxic chemotherapy, its elevation indicates early myocardial injury and dysfunction.^1,7,27,39,42^ Troponin levels increase in children with cardiomyopathies; however, this elevation is not typically correlated with prognosis. It happens particularly in cases of DCM and is often associated with inflammatory conditions like myocarditis.^1,27,39^ In patients with arrhythmogenic cardiomyopathy, troponin may increase in the “warm phase,” as described by Bariani et al., where there is a clinical presentation with chest pain and elevated troponin in the absence of coronary alterations.^43^ The measurement of muscle enzymes and assessment of liver and kidney function are essential for the analysis of multi-organ involvement.^29^
7. Cardiac catheterization: indicated in specific conditions. It determines pulmonary vascular resistance, assesses coronary circulation, rules out coronary anomalies, confirms the physiology of RCM and performs endomyocardial biopsy.^1,3,27,33^

#### Step 2

Step 2 consists of identifying the etiology. Although the morphofunctional phenotype resembles that seen in adults, the prevalence of etiologies differs between children and adolescents. Neuromuscular, metabolic, mitochondrial diseases and other genetic syndromes are major causes, especially in infants and preschool children.^1,2,7,25,29^

Some conditions in childhood are clarified with metabolic investigation and muscle enzyme measurement. Elevated creatine phosphokinase levels may suggest mitochondrial disease and Danon disease in patients with HCM. In contrast, in patients with DCM, elevated muscle enzymes are associated with dystrophinopathies, sarcoglycanopathies, laminopathies, myotonic dystrophy, and desminopathies. Lactic acidosis and elevated transaminases are indicative of mitochondrial disease, while patients with Fabry disease may present with proteinuria.^31,37^

IEM account for approximately 15% of the known causes of cardiomyopathies, and they are common etiologies of HCM and DCM.^2,7,25,32^ Examples include glycogen storage diseases (Pompe disease and Danon disease), lysosomal storage diseases such as mucopolysaccharidoses (MPS), and Fabry disease, which are disorders of glycosphingolipid metabolism.^32^

Neonatal screening protocols for IEM may not include all necessary diagnostic tests, potentially leading to underdiagnosis of IEM-associated cardiomyopathies due to limited awareness.32 Pompe disease should be ruled out in infants with HCM accompanied by hypotonia by measuring the activity of the acid alpha-glucosidase enzyme. If Fabry disease is suspected, the alpha-galactosidase A enzyme should be measured. For MPS, it is important to measure urinary glycosaminoglycans, while Danon disease is caused by a deficiency of lysosome-associated membrane protein 2 (LAMP2).^1,2,7,32^ The etiological diagnosis of this group of diseases is essential due to the possibility of specific treatment, requiring a multidisciplinary approach.^25,38,44^

Noonan syndrome is the main genetic cause of HCM in children under one year and is accompanied by a high risk of early mortality.^1,32^

Genetic evaluation consists of a systematic approach, including a detailed family history analysis (STEP 1), genetic counseling, and specific genetic tests when indicated.^1,42^

The clinical geneticist rules out syndromic conditions and indicates which genetic tests should be requested. It is essential to understand the ethical and legal aspects that vary in each country and the psychosocial impacts that may be generated. Thus, these issues should be discussed during counseling, where the geneticist will also indicate which family members would be at risk of developing the disease, guiding the cascade of clinical and genetic screening of these relatives. The current consensus recommends that genetic investigation be performed on children and adults diagnosed with cardiomyopathy.^37,45-49^

#### Genetic tests

During genetic counseling, the family is informed of the possible results of this investigation: (1) conclusive result, where a pathogenic or probably pathogenic variant is found that justifies the phenotype in question; (2) negative result, where no variant is detected that justifies the cardiomyopathy, or (3) inconclusive result, where an identified variant is classified as a variant of uncertain significance (VUS). In this case, it is important to continue monitoring and reassessing the pathogenic potential of the variant in the future.^1,42,50,51^It is essential that the interpretation of these findings be performed by a professional with training in cardiogenetics.

The likelihood of a positive genetic test result depends not only on the type of cardiomyopathy being evaluated but also on specific clinical features that may suggest associated diseases, such as muscular dystrophies. Thus, the positivity rate of genetic testing can range from 60% to 70% for HCM, 30% to 40% for DCM, and 50% to 60% for arrhythmogenic right ventricular cardiomyopathy in adult patients.^52^In pediatric cardiomyopathies, the determination of genetic etiology occurs in approximately 32% to 39% of cases.^6,53^

Most cardiomyopathies involve monogenic alterations, and next-generation genetic sequencing (NGS) is recommended. Specific panels are available for certain phenotypes. These tests are indicated for individuals who present a well-defined and isolated phenotype of cardiomyopathy. However, in cases of multisystem involvement, dysmorphisms or suspected syndromic cardiomyopathy, complete exome analysis is recommended. The Sanger technique is used for investigating already identified variants in family genetic screening cases.^49,50,54^

Rarely, chromosomal alterations may be associated with cardiomyopathies, such as Pallister-Killian syndrome, a 12p tetrasomy that may occur with HCM, and RCM with an anomaly of chromosome 6. In these cases, the preferred diagnostic test is the peripheral blood karyotype.^55,56^

The high cost of genetic tests and the need for professionals trained in cardiogenetics to indicate and adequately interpret the results limit their use. Moreover, with the widespread use of the exome, secondary or incidental findings can be detected that are not related to the cardiovascular disease being investigated, such as pathogenic variants in genes related to the development of cancer. It is crucial to inform patients about the potential outcomes of genetic testing beforehand, and patients must consent to receive or decline such results.^51^

#### Family screening

This process involves evaluating individuals from the proband’s family, whether they exhibit clinical symptoms or not, due to a family history of a previously defined cardiomyopathy. Those with an autosomal dominant inheritance pattern have a 50% chance of transmitting the variant to their children.^37,50,57^

Clinical screening is advised for all first-degree relatives who are at risk of developing cardiomyopathy, even if they are asymptomatic. Therefore, an evaluation including anamnesis, physical examination, electrocardiogram and color Doppler ultrasound should be performed the identify the phenotype. The Heart Failure Society of America recommends annual clinical screening for children up to age 5, screening every 1 to 2 years for children aged 6 to 12, and screening every 1 to 3 years for adolescents aged 13 to 19. Screening should continue into adulthood, with regular evaluations every 3 to 5 years.^50^

Genetic Screening: once a pathogenic variant is identified, it can be further investigated through cascade screening in first-degree relatives. If the variant is detected, the individual should continue clinical evaluations to identify the phenotype and undergo genetic counseling. A positive genotype does not guarantee clinical manifestation of cardiomyopathy, as penetrance can vary, and different phenotypes can arise from the same variant, illustrating variable expressivity. If the variant is not identified in the studied relatives, there is no need to maintain phenotypic screening with routine cardiovascular exams.^37,50,57^

Parents and guardians often express concerns about potential stigma, discrimination, and psychological impact on their children if they test positive for a family genetic variant. However, the benefits of knowing the genotype are significant and include identifying children who require cardiac care, providing guidance on participation in sports activities, and reducing anxiety when the test results are negative.^50,57,58^

#### Step 3

At this stage, the morphofunctional phenotype and etiology are already defined. Based on this information, the MOGE(S) classification can be employed.^1,9^

#### Step 4

Next, prognosis determination is essential. This varies and is directly related to phenotype, genotype, patient age, presence of cardiac arrhythmias, elevation of biomarkers, and functional class.^1,2,35^

DCS has a poor prognosis, with approximately 30% of patients dying or requiring heart transplantation within three years of the disease due to advanced HF or arrhythmias.^2,34,39^Studies indicate that age at diagnosis (under 1 year and over 12 years), changes in LV systolic and diastolic functions, and LV end-diastolic diameter are predictors of disease progression, adverse events, heart transplantation, and death. Increased NT-proBNP/BNP levels are also associated with worse outcomes.^35,39,44,59-61^ Survival without the need for heart transplantation can range from 60% to 75% within 5 years after diagnosis.^7,33,60^ One third of patients can recover ventricular function, occurring more frequently in patients with biopsy-proven myocarditis, with smaller LV end-diastolic dimension and thicker septal wall. DCM is the primary indication for heart transplantation in children, with a favorable survival rate of 94% in the first year post-transplantation.^1,2,7,39,62^

HCM has a poorer prognosis in children during the first year of life, particularly when caused by inborn errors of metabolism and malformation syndromes.^1,2,7,63^ The presence of malnutrition and HF at diagnosis worsens the prognosis, as evidenced by data from the North American Pediatric Cardiomyopathy Registry.^63^

Survival rates among patients with HCM may vary depending on the underlying etiology. The lowest rates are associated with inborn errors of metabolism (42% at 5 years), followed by malformation syndromes (74%). When the diagnosis is made after one year of age, and the cause is not determined, survival reaches 94% at five years.^2,7,63^

The main cause of death in children with HCM is SD, which is more common than in adults.64 The main risk factors associated with SD are unexplained syncope, maximum LV wall thickness, LA diameter, LV outflow tract gradient, and non-sustained ventricular tachycardia. A family history of SD does not appear to be associated with an increased risk in childhood.^65,66^

Data from the ShaRe (The Sarcomeric Human Cardiomyopathy Registry) study revealed that the presence of sarcomeric variants in children with HCM increases the risk of developing ventricular arrhythmias and necessitating advanced HF treatment. It is associated with a 67% high risk of cardiac outcomes, with a two-fold increased risk of developing HF.^67^ Variants in the MYBPC3 and MYH7 genes are related to malignant arrhythmias.^64^ Risk calculators for the pediatric population are being validated for use in children under 16 years of age (*HCM Risk-Kids* https://hcmriskkids.org/).^36,66^

Patients with the restrictive cardiomyopathy (RCM) phenotype are characterized by the worst prognosis among all cardiomyopathies. The 5-year survival rate is 68%, and the occurrence of HF and reduced shortening fraction complicate the prognosis. They are generally referred to heart transplant programs early.^1,2,7^

In NCCM, prognosis is more closely associated with clinical presentation than with phenotype. Asymptomatic patients with isolated phenotypes of NCCM have better outcomes.^1,2,7,41,68^

#### Step 5

In this final stage, we should explore available treatments for pediatric cardiomyopathies, considering the application of precision medicine.

Individualized patient and family treatment becomes feasible following confirmation of the phenotype. For patients diagnosed with HCM and arrhythmogenic cardiomyopathy, competitive physical activities are restricted, and consideration for implantable cardioverter defibrillator (ICD) placement as primary prevention against sudden death is warranted. Identifying the patient’s genotype and its correlation with the natural history and progression of the disease enables crucial therapeutic decisions, such as prioritizing heart transplantation or initiating enzyme replacement therapy (ERT).^1,2,6,7,29,32,50^

Several IEMs are now treatable with therapeutic strategies targeting their pathophysiology, thus offering the potential for cardiomyopathy reversal in certain cases.^2,7,23,32^In systemic carnitine deficiency, for example, replacing high doses of carnitine improves DCM.^32^

The development of specific therapies has progressed due to advances in clinical research. ERT performed in Pompe disease improves myocardial hypertrophy; the earlier specific treatment is initiated, the better the results.^2,7,23,32^ Other lysosomal storage diseases associated with cardiomyopathy, including Gaucher disease, Fabry disease, and mucopolysaccharidosis I, II, IV, and VI, are treated with ERT or bone marrow transplantation with good results. ERT has also been successfully used for the PRKAG2 mutation.^7,32,38,69^

Early initiation of ICD therapy may be advantageous when mutations in high-risk genes such as DES, SCN5A, FLNC, and LMNA are identified. This is crucial as SD could potentially manifest as the initial symptom before the onset of cardiomyopathy.^35^

The decision to implant an ICD is still a challenge among pediatric patients. There is a risk calculator available (*HCM Risk-Kids* https://hcmriskkids.org/) that can help in the individualized assessment of these patients.^35,66^

Myosin modulators are a new class of pharmaceutical agents under development aimed at treating patients with a variety of cardiomyopathies. A strategy to decrease the myosin ATPase rate has recently proven to be effective for the treatment of obstructive HCM in adult patients, the drug mavacamten.^70,71^

Gene therapy aims to treat the etiology at the molecular level. Bains et al.^72^ recently published a review on its application in monogenic cardiovascular diseases, including cardiomyopathies. Gene editing, silencing and replacement are also possible.

Gene replacement therapy has therapeutic applications when there is a variant with function loss. Thus, the objective is to introduce the functioning gene into the cell through a vector, usually adenovirus. Currently, a clinical trial is testing this type of treatment for cardiomyopathy caused by a pathogenic variant in LAMP2, indicative of Danon disease. Mutations in FLNC, MYBPC3, TTN, DSP, PKPs, BAG3 and LMNA are potential examples to which this therapy could be applied.^71,72^

Gene silencing therapy aims to reduce the expression of a gene containing a pathogenic variant that leads to the production of an altered protein. Specific treatments for Duchenne muscular dystrophy and transthyretin amyloidosis (ATTR) have produced promising results.^71,72^

Gene editing occurs using CRISPR-Cas9 technology, enabling targeted DNA cleavage at a precise location in the genome, determined by the unique sequence of a guide RNA, correcting the mutation present in the gene.^72^

Advances in translational and precision medicine enable the treatment of cardiomyopathies very closely to the genetic etiology, correcting the cause or cellular functional alterations. These therapies have promising applications; however, they are still expensive, which limits their use.^73-76^

## Conclusion

Cardiomyopathies diagnosed during childhood and adolescence form a highly intricate group characterized by diverse etiologies, phenotypes, clinical presentations, and prognoses. A systematic approach is crucial to standardize clinical assessment, thereby ensuring identification of the morphofunctional phenotype, thorough investigation into the etiology using available methods, accurate patient classification for prognosis determination, and personalized application of current therapies tailored to each case.

This structured approach underscores the significance of understanding etiology in shaping personalized treatment plans, ultimately leading to improved prognostic outcomes. Thus, we have developed a systematic five-step approach that integrates the MOGE(S) classification, emphasizing the pivotal role of clinical geneticists.

Exploring the impact of integrating genetic testing for cardiomyopathies within the Brazilian Unified Health System (SUS) has the potential to significantly advance patient care. Therefore, ongoing clinical research in this field remains essential.
